# Stage 1 registered report examining agency and outcome valence on inter-brain synchrony during dyadic moral judgments

**DOI:** 10.3389/fpsyg.2025.1710844

**Published:** 2026-02-12

**Authors:** Neal Hinvest, Nathan Taylor

**Affiliations:** Department of Psychology, University of Bath, Bath, United Kingdom

**Keywords:** ability to affect outcomes, affect, agency, agency attribution, agency manipulations, analysis pipeline, asymmetric agency roles, brain networks

## Abstract

Inter-brain synchrony (IBS) has emerged as a promising marker of neural processes supporting social interaction, cooperation, and moral evaluation. Previous hyperscanning research shows that IBS increases during tasks involving joint attention, cooperation, or shared intentionality. Nevertheless, little is known about how agency—the extent to which individuals can influence outcomes—and outcome valence interact to shape neural alignment between people. Real-world social contexts often involve asymmetries in control and responsibility, and these dynamics may critically influence how individuals coordinate and evaluate one another’s decisions. The present registered report outlines two experiments designed to examine the relationship between agency and outcome valence and IBS. In the first experiment, agency (high vs. low) and outcome valence (reward vs. punishment) are jointly manipulated during moral adjudications of economic game offers. We predict that high-agency contexts, especially when linked to punishing unfairness, will enhance IBS by fostering shared responsibility and coordinated evaluation. In the second experiment, asymmetric agency roles (equal, unilateral, none) are introduced to examine whether unequal distributions of control reduce IBS. We also explore directional influences from higher- to lower-agency partners. By systematically manipulating both the degree of agency and the valence of social outcomes, this research aims to advance theoretical accounts of joint moral decision-making and hierarchical social interaction. The findings will contribute to understanding how responsibility, fairness, and power dynamics shape neural alignment that underpins cooperative and moral behavior.

## 1 Introduction

This study has been pre-registered on the Open Science Framework (OSF; https://osf.io/shqv6), including hypotheses, design, data preprocessing criteria, and planned analyses.

The ability to align with others in joint decision-making is foundational to human cooperation and moral reasoning. Inter-brain synchrony (IBS), the alignment of neural activity between individuals, offers a window into the shared neural dynamics underlying these processes (Balconi and Vanutelli, 2017; Cui et al., 2012). Prior studies have reliably found that IBS increases during cooperative behavior, joint attention, and mutual engagement, particularly in tasks involving shared intentionality or affect (Czeszumski et al., 2020; Dumas et al., 2010; Koike et al., 2015; Valencia and Froese, 2020). Increased IBS is observed in effective clinical relationships and is associated with reduced client symptom severity (Azhari et al., 2025; Sened et al., 2025), within productive and creative work teams (Réveillé et al., 2025; Réveillé et al., 2024), and within effective learning in educational settings (Dikker et al., 2017; Tan et al., 2023). Research into neural synchrony advances understanding from within single- to between-mind interactions, developing insights into the neuroscience of social interactions in more typical interactive experiences (Babiloni and Astolfi, 2014; Carollo and Esposito, 2024; De Felice et al., 2025; Kasai et al., 2015).

Recent theories posit that social interaction involves adaptive, dynamic shifts between inter- (synchronization) and intra- (segregation) personal interactive states (Gordon et al., 2025; Mayo and Gordon, 2020) and that IBS reflects these dynamic shifts (Bilek et al., 2022; Froese et al., 2024). A recent study by the author and colleagues has provided further nuance to the theory. Hinvest et al. (2025) measured IBS in dyads interacting in a naturalistic conversational paradigm in which participants identified violations of injunctive norms and decided upon joint actions. Through linguistic analysis, sections of their conversation were mapped to stages of shared identity formation (Smith et al., 2015a,b). Greater IBS was observed during the formation stage of a shared social identity between the dyads, a link between an individual’s personal identity and the social group to which the individual belongs (Hinvest et al., 2025). These findings align with theories of joint action, which hold that the coordination of action to produce a joint outcome forms the basis for shared intention and action (Knoblich et al., 2011; Tomasello et al., 2005), as well as for the negotiation of joint agency (Pacherie, 2011). Thus, IBS potentially reflects dynamic shifts between states of intrapersonal identity (segregation) and interpersonal identity (synchronization).

The perceived ability to affect outcomes within a situation, or agency, is critical for effective joint action in social interaction (Loehr, 2022; Sebanz et al., 2006). The Comparator Model is the predominant account of sensorimotor agency whereby predicted and observed sensory outcomes are compared to determine self or other agency (Carruthers, 2012; David et al., 2008; Frith, 1992) and has been complemented by higher-order approaches emphasizing metacognitive and contextual factors in agency attribution (Synofzik et al., 2008). Theoretical accounts suggest that agency modulates not only individual cognition (Haggard, 2017) but also shared neural processes (Redcay and Schilbach, 2019). Agency manipulations have been found to typically engage the fronto-parietal and temporal regions, the cerebellum, and the insula (Crivelli and Balconi, 2017; Haggard, 2017; Wakata and Morioka, 2014, 2015). In terms of experimental findings on neural synchrony, perceived joint agency, the extent to which partners feel jointly in control, was found to modulate IBS in a motor synchrony (finger-tapping) task (Shiraishi and Shimada, 2021).

Sun et al. (2019) used the Trust Game, a two-player economic exchange in which one participant (the “trustor”) decides how much money to send to another (the “trustee”), knowing the amount will be multiplied. The second participant then chooses how much to return, to investigate IBS in situations that provoke representations of power vs. trust. Framing the game in terms of power asymmetry, by calling the game “The Power Game,” was found to increase IBS in frontal alpha aligned with more strategic interactive decision-making compared to framing the game in terms of shared outcomes, by calling the game “The Trust Game” (Sun et al., 2019); however, measurement of perceived agency was absent, thus conclusions within this work as to the link between perceived agency and IBS are tentative. While many studies report increased IBS in cooperative or joint-agency contexts, findings are not uniform (Holroyd, 2022). IBS effects can vary by task type and analysis pipeline, and some studies report null or chance-level effects when rigorous controls are applied (Czeszumski et al., 2020; Zimmermann et al., 2024). These mixed findings underscore the need for tightly controlled designs and careful interpretation, which the present study incorporates.

Converging evidence from social neuroscience implicates the dorsolateral prefrontal cortex (dlPFC), medial frontal cortex (mPFC), and right temporoparietal junction (rTPJ) in processes central to agency, including norm enforcement, conflict monitoring, and mentalizing (Haggard, 2017; Sanfey et al., 2003; Siegal and Varley, 2002). While these regions are not exclusively agency-specific, their consistent involvement in joint agency paradigms makes them strong candidates for investigation. Given the volume of theory and observations highlighting the impact of agency on joint motivation and action and the lack of multi-brain studies in this area, this is a fruitful research pathway for understanding the critical role of agency in interpersonal neural synchrony.

Alongside agency, outcome valence represents a second influential determinant of social decision-making and neural coupling. Valence shapes perceptions of fairness and moral evaluation, influencing both individual and interactive neural processes. This is observed in economic exchange games, such as the Ultimatum Game, in which a proposer offers a share of money to a responder, who can accept or reject the offer, leaving both players with nothing. Observation of neural activity within the Ultimatum Game has shown that offers deemed unfair (very heavily weighted in terms of the proposer’s benefit) evoke heightened neural responses in regions implicated in norm violation and moral evaluation in responders (Civai et al., 2012; Gabay et al., 2014; Sanfey et al., 2003). These responses consistently engage the dlPFC and mPFC, reflecting the cognitive control and evaluative conflict involved in processing unfair outcomes (Feng et al., 2015; Gabay et al., 2014), and support their inclusion as regions of interest in studies examining the effects of outcome valence on IBS.

Investigation of third-party judgments of others’ social decisions elucidates psycho-social factors associated with fairness and moral evaluation and how underlying brain networks operate within social dynamics. Research on norm enforcement shows that perceived unfairness evokes strong motivations for punishment, even when enacted by third parties (Buckholtz et al., 2008; Ciaramidaro et al., 2018; Fehr and Fischbacher, 2004), suggesting that outcome valence is central to collective moral evaluation. Third-party punishment variants of the Ultimatum Game reveal a common preference to punish proposers when expected fairness norms are violated (Mattan et al., 2020). Third-party punishment paradigms are associated with brain regions generally associated with social and affective processing (Buckholtz et al., 2008; Ciaramidaro et al., 2018), notably the aforementioned lateral frontal, medial frontal, and temporoparietal regions within the mentalizing system (Siegal and Varley, 2002). More specifically, these regions have been associated with moral evaluation, judgments of others’ mental states, and information integration (Ginther et al., 2016). Most empirical research on third-party punishment has focused on isolated individual judgments (Buckholtz et al., 2008; Fehr and Fischbacher, 2004); however, in naturalistic settings, third-party evaluations often emerge through social interaction, in which deliberation and the attribution of shared responsibility can shape moral outcomes. Neural synchrony paradigms offer a more ecologically valid approach to understanding the neurobiology of shared moral evaluation, a domain that hyperscanning can readily address (Ciaramidaro et al., 2018; Shamay-Tsoory and Mendelsohn, 2019; Sobeh and Shamay-Tsoory, 2025).

Clear, measurable links have been identified between the agency and the valence of the outcome. Agency has been found to be modulated by the valence of action outcomes (Barlas et al., 2018; Barlas and Obhi, 2013; Majchrowicz et al., 2020; Yoshie and Haggard, 2013), typically measured by the intentional binding effect—the perceived temporal compression between a voluntary action and its outcome (Moore and Obhi, 2012). However, intentional binding primarily reflects sensorimotor aspects of agency and may not capture higher-order social dimensions. Extending this work with hyperscanning paradigms allows examination of links between agency and outcome valence in interactive contexts, addressing the traditional ecological limitations of single-person designs.

Social psychological accounts of power and hierarchy indicate that asymmetric distributions of control modulate perceptions of responsibility and alignment (Caspar et al., 2020; Guinote, 2017; Keltner et al., 2003). Joint agency in interpersonal coordination, in line with agreed fairness norms, modulates decision-making behavior (Arvanitis et al., 2019). Theoretical accounts cohesively point toward increases in joint agency and shared moral evaluation being associated with shifts toward interpersonal cognitive and affective synchronization (Knoblich et al., 2011; Loehr, 2022; Pacherie, 2011; Sebanz et al., 2006; Sobeh and Shamay-Tsoory, 2025; Tomasello et al., 2005), aligning with theories positing that the fluctuation between inter- and intrapersonal states underlies social cohesiveness and shared goal orientation and self-orientation (Gordon et al., 2025; Mayo and Gordon, 2020). Together, the frameworks discussed predict that perceptions of both agency and outcome valence are associated with inter-brain synchrony. However, neural synchrony accounts of the effects of agency and outcome valence on joint moral evaluation and decision-making are largely absent, despite both being central to theories of cooperation, responsibility, and morality.

The present study aims to advance theoretical accounts of how distributed control and evaluative outcomes jointly shape shared neural processes in dyads, using two experiments. Experiment 1 examines the factorial influence of agency and outcome valence, while Experiment 2 isolates asymmetric agency. Together, these studies will inform models of moral decision-making and the neural mechanisms by which social hierarchies influence interpersonal alignment.

## 2 Materials and methods

### 2.1 Overview of study design

Two within-subject experiments will use an *f*NIRS hyperscanning paradigm to record simultaneous cortical activation in dyads. Each experiment manipulates agency conditions across a series of moral judgment tasks involving third parties.

### 2.2 Participants

Each experiment includes 30 dyads (60 participants). A power analysis was conducted using G*Power for a 2 × 2 within-subjects repeated-measures design (Agency × Valence), assuming a medium effect size (Cohen’s *f* = 0.20), *α* = 0.05, and power = 0.80. This analysis indicated a required sample size of 30 dyads. This estimate is conservative given the expected medium-to-high reliability of inter-brain synchrony metrics derived from hyper-*f*NIRS (Czeszumski et al., 2020).

To provide further validation, a Monte Carlo simulation was conducted using the *simr* package in R, modeling dyadic random intercepts and realistic trial-level variability. *R* code, including simulation parameters, can be found on OSF. The model was fitted using lmer(IBS ~ Agency * Valence + (1 | dyad)), and power was estimated for the Agency × Valence interaction using powerSim() with 100 simulations. The results confirmed that 30 dyads provide sufficient power (~80%) to detect the hypothesized interaction effect under these assumptions. A further simulation was conducted for the 1 × 4 within-subjects design, again confirming that 30 dyads would provide sufficient power.

Participants must be aged 18–40 years, fluent in English, and right-handed (assessed using a brief version of the Edinburgh Handedness Inventory (Veale, 2013)). The exclusion criteria include a lifetime diagnosis of a neurological/psychiatric disorder, a history of head trauma, or uncorrected visual impairments. Same-sex dyads will be used to account for gender differences, such as empathy or agency perception, and to address potential gender-based asymmetries in power/dominance (especially under hierarchy manipulations). Participants will be unfamiliar with each other prior to the study. They will be recruited via a university participant pool and local advertisements.

Ethical approval will be obtained from the University of Bath Social Science Research Ethics Committee. Participants will receive course credit or be reimbursed up to £20 for their time. Dyads will be age-matched (within 5 years).

### 2.3 *f*NIRS data acquisition

An NIRx NIRSport2 *f*NIRS system will be used in a dual-headset configuration. A sampling rate of 10.2 Hz will be used, as it enables reliable IBS analysis without compromising signal fidelity or synchronization while minimizing unnecessary data burden (Zhang et al., 2020; Zhang et al., 2024). Thirty-two optodes (16 sources × 16 detectors) were divided between two participants, yielding 16 per participant. One detector per participant was allocated to short channels.

**Figure 1:**
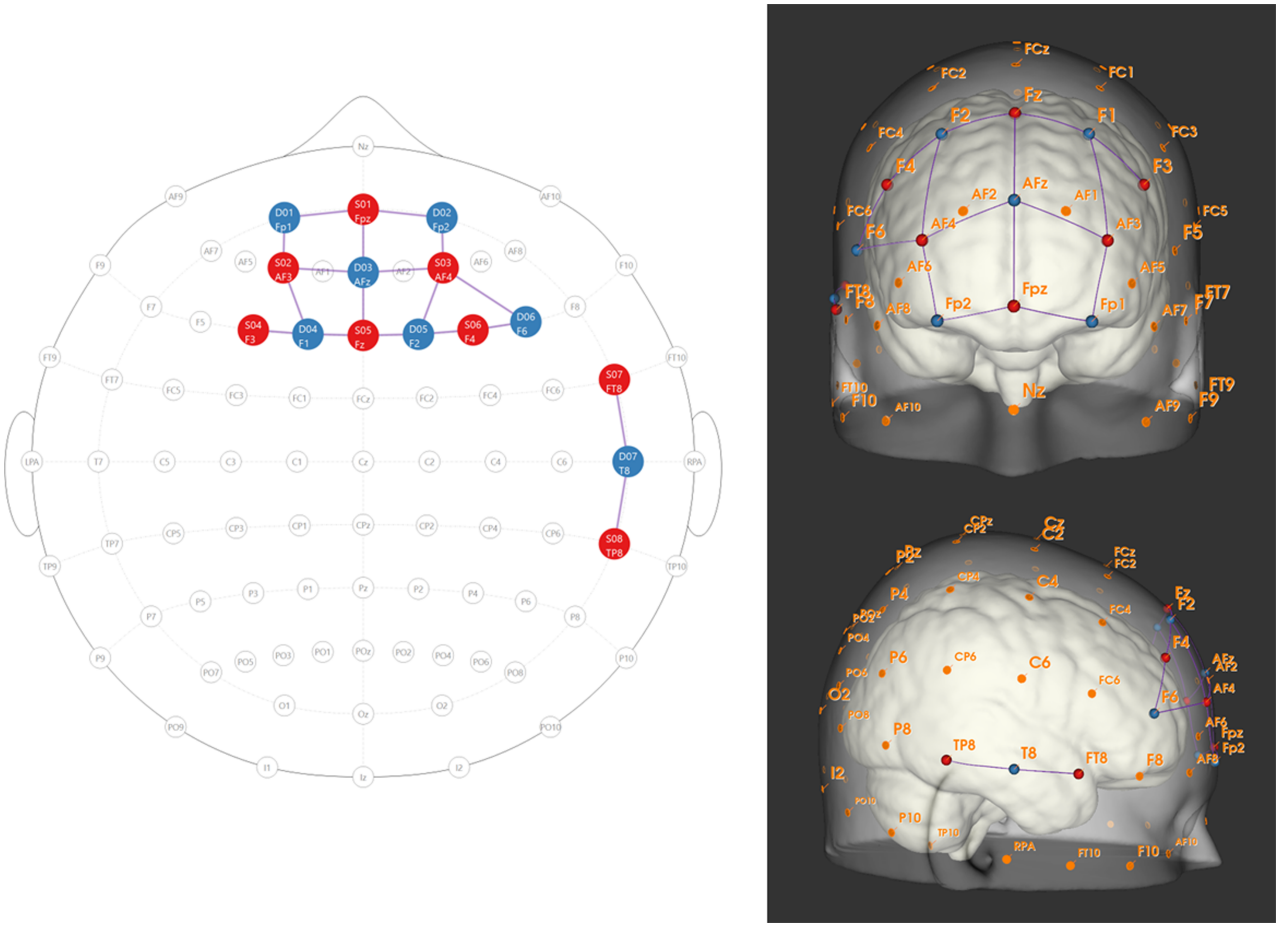
Schematic of optode placement based on the international 10–10 system visualised using NIRSite (https://nirx.net/nirsite). Red circles represent source optodes; blue circles represent detector optodes. Source–detector distances were maintained at approximately 35 mm to optimize cortical sensitivity. Regions of interest (ROIs) include the dorsolateral prefrontal cortex (dlPFC; optodes positioned at, and neighbouring to, F3, F4, F6), medial prefrontal cortex (mPFC; optodes at, and neighbouring to, Fpz and AFz), and right temporo-parietal junction (rTPJ; optodes at FT8, T8 and TP8). Note that one detector (per participant) is used for short channels. The left panel shows the 2D layout of optodes on the 10–10 system, with connecting lines indicating source–detector pairings that form measurement channels. The right panel shows the same optode positions projected onto a standard head model (MNI space) from frontal (top) and right lateral (bottom) views, illustrating anatomical correspondence with target cortical regions. This montage was designed to maximize coverage of the specified ROIs while maintaining recommended inter-optode spacing for adult participants.

The fNIRS Optodes Location Decider (fOLD) was used to spatially locate the regions of interest using a 10–10 mapping (Zimeo Morais et al., 2018). Figure 1 shows a schematic of the locations of the optode sources and detectors. These regions were selected for their well-documented roles in social processing and decision-making within the UG (Carrington and Bailey, 2009; Gabay et al., 2014; Siegal and Varley, 2002).

### 2.4 Task paradigm

#### 2.4.1 Common elements in both experiments

Participants will make third-party adjudications of ultimatum game (UG) offers, using an existing third-party adjudication game implemented in a hyper-scanning protocol (Ciaramidaro et al., 2018). The UG is a two-player economic game in which one player (the proposer) is given a sum of money and must offer a portion of it to a second player (the responder). The responder can either accept the offer, in which case both players receive the proposed amounts, or reject it, in which case neither player receives anything. The UG is widely used to investigate fairness, norm enforcement, and moral judgment.

Participants will make private, non-verbal responses throughout both experiments. This approach minimizes motion artifacts associated with speech. It avoids introducing additional cognitive and social processes (e.g., language production, negotiation) that could confound hyperscanning measures of fairness and agency (Jahng et al., 2017). Non-verbal input (via keyboard or button press) ensures experimental control and consistent timing across trials, supporting clean interpretation of inter-brain synchrony. Our design choice settled upon the standard, rather than the iterative UG task (Shaw et al., 2018), as the iterative version introduces strategic complexity that could confound the effects of agency and outcome valence on neural synchrony. Moreover, the standard UG provides a consistent and interpretable framework for isolating moral evaluation processes, which is essential for time-locked analysis of inter-brain synchrony using *f*NIRS.

#### 2.4.2 Experiment 1: (2 × 2 design)

Participants will be presented with a range of fair/unfair offers within the UG. They will be informed that the offers were made by human players in an earlier experiment and that (unknown to the players) their adjudication decisions will affect the final payoffs of those prior players. In reality, there were no players; the offers were designed by the researchers. Participants will individually choose an amount of money (−£5 to £5 in unit increments) to influence the proposer’s payout.

**Figure 2:**
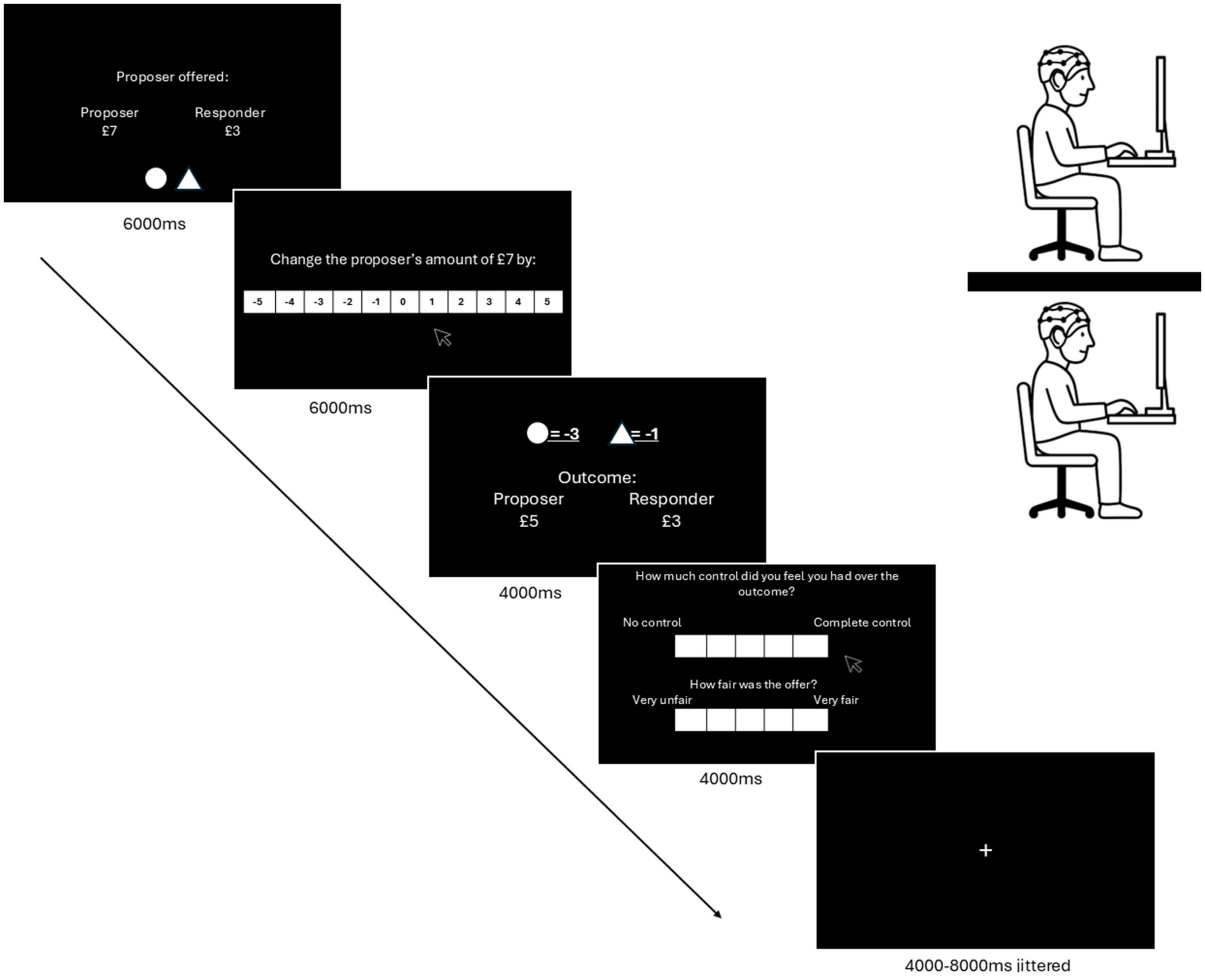
Structure of the experimental task. Participants make non-verbal responses facing their own device, separated from the other player by a wall divider. Screen 1 (offer presentation): Participants are presented with the offer from the proposer to the responder, taken from a prior ultimatum game (participants are not told that these offers are not genuine from that prior game). The agency type is depicted by a circle and a triangle at the bottom of the screen. Each participant is assigned a shape; if that shape is presented, they have agency in their subsequent decision input. In the trial depicted here, both players have equal agency. Screen 2 (decision input): Each participant separately enters an amount of money from −£5 to £5 in unit intervals to reward/punish the proposer. Screen 3 (agency resolution and outcome display): The outcome for the proposer is displayed. In the high-agency condition, both participants’ decisions are presented. In the low-agency condition, both are presented alongside a computer-generated amount beneath them. The outcomes enacted (player or computer) are visually emphasized to highlight which actions affect the proposer’s outcome. Screen 4 (agency perception and fairness rating): Participants separately rate their perceived control over the outcome and their subjective rating of the fairness of the proposer’s offer. Screen 5 (rest): A fixation point is presented with a jittered duration of 4,000–8,000 milliseconds (ms) using a Gaussian kernel.

There are two factors: agency and outcome valence. For agency, participants have either high agency, where their choices are averaged and implemented, or low agency, where their choices are used only for feedback, with the outcome determined randomly by the computer (pre-generated). Outcome valence is manipulated by presenting a fair or unfair offer in the UG. There will be 60 trials per dyad (15 per condition), with order counterbalanced using a Latin Square design. Figure 2 shows the task structure.

#### 2.4.3 Experiment 2: 1 × 4 asymmetry design

Experiment 2 follows the same trial structure as Experiment 1, with the following four conditions: Equal Agency, 2 × Unilateral Agency (one per participant), and Random Generator. In the Equal Agency condition, decisions are averaged across the two participants. In each of the two unilateral conditions, only one participant’s choice is implemented, with the choice differing across the two conditions (the participant whose choice is implemented will be visually emphasized to make agency discrepancies clear; see Figure 2). The fourth condition is the Random Generator, which assigns a random value from the provided options. To simplify the procedure, only unfair trials are presented to participants in a third-party punishment protocol (Ciaramidaro et al., 2018). There will be 60 trials per dyad (15 per condition), with conditions counterbalanced using a Latin square design.

### 2.5 Data pre-processing

Data will be pre-processed using Satori,^1^ which provides standardized *f*NIRS workflows with full parameter logging, and subsequently exported to MATLAB/Python for IBS and directional connectivity analyses. Raw intensity data will be converted to optical density and then to HbO/HbR concentration changes using the modified Beer–Lambert law (Scholkmann et al., 2010). Wavelet-based filtering (Molavi and Dumont, 2010) and spline interpolation will be applied to reduce motion artifacts. A band-pass filter (0.01–0.10 Hz) will retain the hemodynamic response while excluding slow drifts and high-frequency noise. Short-separation channels and auxiliary physiological signals (PPG/SpO₂, respiration, and EDA) will be included as nuisance regressors to control for extracerebral and systemic artifacts. As an additional check, surrogate-pair analysis (randomly pairing participants within dyads) will be conducted to estimate synchrony due to chance and shared environments, ensuring that observed IBS reflects genuine interpersonal coupling. Physiological signals will be recorded using the NIRxWINGS system. Channels with a poor signal-to-noise ratio (coefficient of variation > 15%) will be excluded; dyads with >20% excluded channels will be removed. Satori automatically documents the parameters chosen at each step (these processing logs are archived on OSF for transparency).

The following protocols will be implemented to ensure data quality. First, participants will be removed from analysis if they have >20% of trials with response times <200 ms. Second, >20% of identical consecutive responses will trigger review. Finally, in random generator trials or unilateral agency, when the participant’s chosen outcome is not enacted, those with a reported mean perceived agency >4 will be excluded from the analysis. In trials with participant agency, participants with a reported mean perceived agency <2 will be excluded. All exclusions will be reported in CONSORT-style flow diagrams in the Supplementary material and on OSF.

### 2.6 Statistical analysis

Across both experiments, IBS will be compared within regions of interest (ROIs). ROIs have been identified in the literature that posit the role of these areas in social and reward-related evaluation and in their suitability for measurement with *f*NIRS. This ROI-based approach embeds strong *a priori* predictions, tightly links hypotheses to existing theoretical models of neural function, limits the number of comparisons (reducing the risk of type I error), reduces the risk of p-hacking, and improves replicability. *A priori* selected ROIs are the dorsolateral prefrontal cortex (dlPFC), medial frontal cortex (mFC), and right temporoparietal junction (rTPJ). The selected ROIs reflect a convergence of evidence from social neuroscience, economic decision-making, and moral psychology and are hypothesized to show condition-specific modulation of IBS in response to variations in perceived agency and outcome valence (Buckholtz et al., 2008; Gabay et al., 2014; Haggard, 2017).

Wavelet Transform Coherence (WTC) will be computed for homologous ROI pairs across dyads (Grinsted et al., 2004). WTC is the most widely used method for *f*NIRS-based IBS measurements (Cui et al., 2012; Hakim et al., 2023; Nazneen et al., 2022). Although this study is not a direct replication, the design includes internal replication across two experiments with matched procedures. Additionally, repeated measures and dyadic modeling enhance the robustness of the findings. All materials, code, and data will be made available via OSF to facilitate independent replication.

For both experiments, condition-wise IBS values will be averaged across each ROI pair within the dlPFC and mFG. IBS values will be computed for the rTPJ in isolation, as it is unilateral. IBS values will be entered into repeated-measures ANOVAs (described in the next two paragraphs). Multiple comparisons across ROIs will be controlled using the false discovery rate (FDR) at *q* < 0.05 (Benjamini and Hochberg, 2018).

For Experiment 1, a 2 × 2 repeated-measures ANOVA will be employed. It is hypothesized that, within the Decision Input event (screen 2 in Figure 2, event timing 6,000 ms), high-agency trials will elicit greater IBS in the dlPFC and mFG, reflecting shared cognitive engagement in moral deliberation. An interaction is expected, with the strongest IBS under high-agency and punishment conditions.

For Experiment 2, a repeated-measures ANOVA across four agency conditions will be conducted on the Decision Input event (screen 2 in Figure 2, event timing 6,000 ms). We hypothesize that IBS will be greatest in the full agency condition, moderate in the single agency condition, and lowest in the no agency condition. Planned pairwise contrasts with Bonferroni correction will be conducted to test whether there is a significant difference in mean IBS between conditions.

Exploratory analyses in Experiment 1 will focus on the Offer Presentation (6,000 ms) and Outcome Display (4,000 ms) events. During the Offer Presentation, we will examine whether, compared to fair offers (which are likely to elicit punishment), unfair offers are associated with greater IBS in the rTPJ, irrespective of agency, reflecting a shared evaluation of norm violations. During Outcome Display, we will explore whether high-agency trials, compared to low-agency trials, are associated with increased IBS in the rTPJ, particularly following punishment outcomes, indicative of shared affective processing.

In Experiment 2, directional coupling will be assessed using Granger causality and transfer entropy (Bastos and Schoffelen, 2015) as exploratory analyses to test whether asymmetric agency induces directional influences from high- to low-agency participants.

## Data Availability

The original contributions presented in the study are publicly available. Datasets created through this study will be available via the Open Science Framework at https://osf.io/shqv6.

## Footnotes

1
https://nirx.net/satori
